# Research Progress of High-Temperature Resistant Functional Gel Materials and Their Application in Oil and Gas Drilling

**DOI:** 10.3390/gels9010034

**Published:** 2022-12-30

**Authors:** Junwei Fang, Xiong Zhang, Liang Li, Jianjun Zhang, Xin Shi, Guangqiang Hu

**Affiliations:** 1 Petroleum Engineering Technology Research Institute, SINOPEC Northwest Oilfield Company, Urumqi 830011, China; 2 Key Laboratory of Enhanced Recovery for Fracture-Cave Oil Reservoir, SINOPEC, Urumqi 830011, China

**Keywords:** functional gel, high temperature resistant, lost circulation materials, temporary plugging, gel breaking method

## Abstract

With the development of oil exploration, the number of complex situations encountered in the drilling process is continuously increasing. During the operation of large displacement and horizontal wells, the safe density window of drilling fluid is narrow in complex formations and the lost circulation problem is becoming increasingly prominent. This can easily cause the drilling fluid to enter the formation from inside the well through lost circulation channels, which will prolong the drilling cycle, increase drilling costs, affect geological logging, and could cause a series of malignant accidents (such as blowout, sticking of a drilling tool, borehole collapse, and well abandoned). According to the severity, common lost circulation can be classified into three types: fractured lost circulation, karst cave lost circulation, and permeability lost circulation. Currently, researchers are developing different types of lost circulation materials (LCMs) for various lost circulation situations. Compared with conventional lost circulation control methods, the polymer gel lost circulation control technique applies a three-dimensional cage-like viscoelastic body formed via the crosslinking reaction of polymer gels. These materials have strong deformability and can enter fractures and holes through extrusion and deformation without being restricted by lost circulation channels. They then settle in the lost circulation formation and form a plugging layer through a curing reaction or swelling effect. Among the polymer gel LCMs, high-temperature resistant polymer gels can either be used alone or in combination with other LCMs, bringing the advantages of adjustable gelation time, strong lost circulation control ability, and strong filtration ability of the plugging slurry. Moreover, they are suitable for the lost circulation control of microporous leaky layer and have limited influence on the performance of drilling fluids. Therefore, the high-temperature resistant polymer gel lost circulation control technique is increasingly becoming a hot spot in the research of LCMs nowadays. This paper summarizes the research progress into high-temperature resistant functional gels for profile control and water shutoff, lost circulation prevention and control, and hydraulic fracturing. Furthermore, the current application status of high-temperature resistant gels and high-temperature resistant gel temporary plugging agents is demonstrated, followed by a detailed overview of the gel-breaking methods. Overall, this research lays the theoretical foundation for the application and promotion of high-temperature resistant gels.

## 1. Introduction

Lost circulation is a phenomenon in which a large amount of drilling fluid leaks into the formation during the drilling process, resulting in significant drilling fluid loss and prolonged drilling cycles. Moreover, if handled improperly, lost circulation can also cause complications, such as borehole collapse, blowout, and sticking of a drilling tool [1]. Ultimately, lost circulation can even lead to wells being abandoned and major engineering accidents. According to the severity, lost circulation can be classified into three types: fractured lost circulation, karst cave lost circulation, and permeability lost circulation. Currently, lost circulation, especially malignant lost circulation in fractured formations, has not been resolved effectively [2]. This results in increased drilling time and economic losses and has become a worldwide problem that is limiting the development of oil and gas exploration.

Regarding the theory and technology for lost circulation control in fractured formations, based on the classical “stress cage” theory, researchers worldwide have successively proposed the theories of “fracture tail sealing”, “permeability zone”, and “strength ring” [3]. Subsequently, a series of drilling fluid pressure-bearing lost circulation control techniques were developed based on these theories. Although these have achieved satisfactory application results for small-scale fractured lost circulation formations, the success rate of one-time plugging is still low for malignant fractured lost circulation formations [4]. Moreover, the lost circulation control methods are difficult to replicate. Blindness and unpredictability remain when applying most gel lost circulation materials (LCMs) for drilling fluid lost circulation control and prevention in complex formations [5]. There is also a lack of scientific and targeted countermeasures for lost circulation control, and problems such as poor adaptability to lost circulation formations, poor temperature resistance, and insufficient pressure-bearing and resident capacities of LCMs remain unresolved [6].

For low-temperature reservoirs with a high mineralization degree, polymer gel water shutoff agents have disadvantages such as slow gelation, low gel strength, and poor gel stability. Zhang et al. optimized the formulation of an acrylamide monomer polymer gel water shutoff agent and readjusted the optimal concentrations of monomer, crosslinker, initiator, and retarder [7]. This optimized water shutoff agent exhibited improved gel strength, controllable gelation time, enhanced stability, and good shear resistance and was able to form a stable plugging layer under high-mineralization conditions.

Currently, gel-breaking methods include environmental (high-temperature or high-salinity environments) self-breaking, oxidizing breaking, and acid-etching breaking [8]. Among these, self-breaking time is poorly controllable, and oxidizing/acid-etching breaking requires subsequent auxiliary procedures. Commonly used gel-breakers include oxidants, enzymes, and acids. Oxidants are frequently used for gel breaking, the speed of which depends on the decomposition rate of the oxidant [9]. Oxidants decompose relatively slowly in low temperatures and excessively quickly in high temperatures, resulting in an uncontrollable gel breaking rate, risking desanding due to fast gel breaking. By acting as catalysts, enzymes are not consumed in the breaking reaction and can maintain a high breaking level of polymers [10]. However, they lose their activity under high-temperature conditions. Most of the acids used as gel breakers are organic acids. They affect the solution pH when dissolved, where the rate of pH change is determined by the initial buffer concentration, reservoir temperature, and acid concentration [11]. In operation practice, oxidants, enzymes, and acids all have corresponding problems, and suitable gel breakers need to be selected according to environmental conditions, including temperature, pressure, and pH in the reservoir.

To improve the high-temperature stability of polymer gel materials to fulfill the requirements of temporary plugging in high-temperature high-salinity reservoirs and enhance the recovery of crude oil, the performance of existing temporary plugging agents must be significantly improved and optimized. The current research mainly focuses on preparing high-temperature resistant polymers and crosslinkers, as well as filling the gel structure with high-temperature resistant solid materials. In the future, in-depth research and development of novel high-temperature resistant polymers and crosslinkers from the perspective of molecular design are required to provide a reference for further preferential selection of solid filling materials with excellent performance.

## 2. Research Progress of Functional Gel Materials and Their Applications

Currently, polymer gel LCMs are mainly being developed by the three oilfield services giants (Schlumberger, Baker Hughes, and Halliburton), while research on polymer gel LCMs in China started at a relatively late time in comparison [12]. Several downhole cross-linking polymer gel plugging agents were developed with acrylamide-type monomers as the core materials that are crosslinked by inorganic or organic crosslinkers. However, the highest temperature resistance can only reach 140 °C, which does not fulfill the requirements for deep drilling lost circulation control. Table 1 details the characteristics of gel products of several oil service companies and institutes in different countries.

### 2.1. Research Progress of Gels for Profile Control and Water Shutoff Purposes

The application of profile control techniques can effectively control the oil–water ratio and adjust the water injection profile in the water injection layer, significantly improving the recovery rate of oil fields. However, the increasing exploitation of high-temperature high-pressure reservoirs presents more requirements for profile control agents. The emergence of novel materials (such as nanomaterials) also opens a new development path for gel profile control agents (Table 2). In situ chemical crosslinked polymer gels were the earliest gel systems to be applied in oil and gas drilling. To address the different complex reservoir environments, three main gel systems were developed: monomer crosslinked gels, metal crosslinked gels, and organic crosslinked gels. Along with the development of technology and new materials, ground chemical pre-crosslinked gels have emerged (mainly including pre-crosslinked gel particles and polymer gel microspheres), which have great development potential.

In situ crosslinked polymer gels have low viscosity before gelation, allowing them to penetrate deep into the formation and enter small fractures. For deep profile control, based on permeable polymeric network gels, Fang et al. delayed the crosslinking time by adding retarders, providing the gel solution with more migration time in the formation, allowing them to enter the deep part of the formation before gelation [11]. However, mixed gel solutions are prone to filtration loss when injected into the formation, reducing the amount of gel. Accordingly, secondary crosslinking techniques were developed. For example, the gel water shutoff agent prepared using hydrolyzed polyacrylamide (HPAM) by Prifer Inc is highly hydrophilic and can preferentially enter high permeability layers for water shutoff [21]. Subsequently, it breaks upon adding a gel breaker to restore the permeability, effectively avoiding damage to the formation. Multiple field applications have been conducted in the United States, New Mexico, and Canada, where the water content declined by 94% (on average) for all types of reservoirs, with an average enhanced oil recovery of 94%, significantly improving the recovery rate.

Ground pre-crosslinked gel particles for profile control can be retained or adsorbed after entering the permeable layer, becoming trapped in pore throats or macropores to form bridging [22]. The phenolic resin dispersed particle gel prepared by Zhao et al. displayed good swelling properties [23]. The particles agglomerated to form larger particles after being injected in the formation and then bridged to plug the high permeability layers. The smaller the size of ground pre-crosslinked gel particles, the longer the migration distance, and the more likely it is to achieve deep profile control. Liu et al. synthesized submicron to micron phenolic crosslinked polymer gels [24]. Here, the dispersed gel particles could plug the high permeability layers by aggregating in large pores or directly plugging small pore throats, and the particles were able to migrate to the reservoir porous media through elastic deformation and achieve deep profile control. To improve the temperature resistance of the profile control agent and alleviate its negative impact on the formation, Han et al. mixed resorcinol with formaldehyde to form phenolic resin as a crosslinking agent [25]. They then used oxalic acid and ammonium chloride as stabilizers to prepare a polymer gel, where the stability of the plugging layer was well maintained under a high-temperature environment of 200 °C. In addition, the gel displayed enhanced plugging ability, compatibleness, and breaking performance.

**Figure 1:**
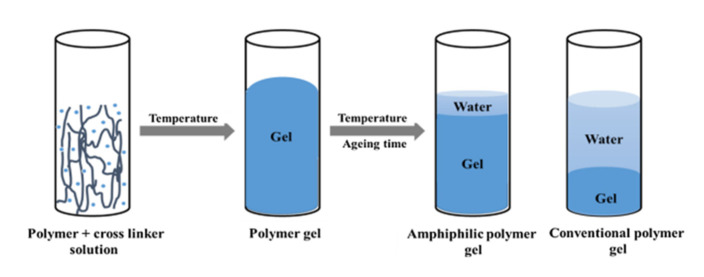
Diagram of the gelation and dehydration process of amphiphilic polymer gel [28].

**Figure 2:**
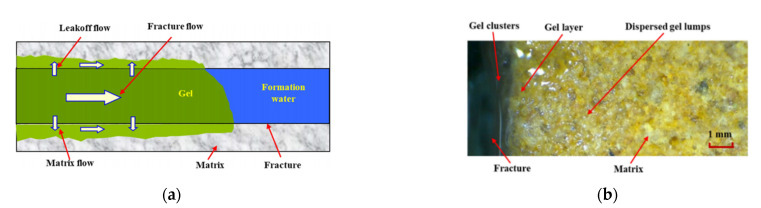
(**a**) Migrating flow of the gel solution in the fracture and matrix, and (**b**) overall distribution pattern of polymer gel in the fracture and matrix after gelation [29].

Profile control and water shutoff are common methods of improving the recovery rates of oilfields by water injection development [26]. Although water shutoff agents have been widely used in oilfields for decades, for low-temperature high-mineralization reservoirs, polymer gel water shutoff agents have disadvantages, including low gel strength and poor gel stability [27]. Yang et al. formulated a new gel system with a low concentration of amphiphilic polymer AP-P4 and an organic chromium crosslinker [28]. The dehydration rate was reduced and the system successfully maintained stability under high-salinity environments (Figure 1). Here, the polymer gel water shutoff agent is presented as gel clusters in the fracture, a gel layer on the fracture surface, and dispersed gel lumps in the matrix pores (Figure 2). Using polymer LH2500, CYJL as the crosslinker, citric acid as the regulator, sodium sulfite as the retarder, and sodium polyphosphate as the enhancer, Huang et al. successfully reduced the initial viscosity of the gel to fulfill the requirements of deep plugging [29]. Enhancing the gel’s plugging ability is fundamentally based on improving the adsorption of gel clusters in the fracture to the fracture surface.

### 2.2. Research Progress of Gels for Lost Circulation Prevention and Control

Materials for lost circulation prevention and control are very important for the effective prevention and treatment of lost circulation under different working conditions. In recent years, researchers have developed various types of these materials, mainly including highly acid soluble, smart, high-temperature resistant, and oil-swelling gel LCMs. Polymer gel LCMs are applicable to different sizes of lost circulation channels and can be compounded with other materials to enhance the viscous resistance [21]. Moreover, the crosslinking time and strength of these gels can be controlled to display different characteristics. Polymer gels have good adaptability to lost circulation channels, low content of the solid phase, resistance to dilution, high flowing plugging capacity, and lubricity. They are also conducive to reducing the occurrence of sticking of a drilling tool and can be divided into pre-crosslinked polymers and non-crosslinked polymers with special structures [30]. Moreover, they can be used in combination with other LCMs.

Polymer gel LCMs enter the lost circulation formation as a liquid or highly viscous fluid before gelation and form a crosslinked polymer in the lost circulation channels [31]. After gelation, the gel adheres to the wall surface of the lost circulation channel via chemical bonds in the polymer and the intermolecular force, producing a large viscous resistance and forming a solidified plugging layer in the lost circulation channel. Water/oil swelling polymer LCMs enter the lost circulation formation under the action of pressure difference and absorb water or oil molecules into the network structure through intermolecular van der Waals forces or hydrogen bonding and osmotic pressure inside and outside the three-dimensional network structure [32]. This causes drastic volume changes and forms adaptive materials with a certain elasticity. The materials can then automatically adjust their shape to fit the size of the lost circulation channel, forming effective plugging.

With regard to pre-crosslinked polymer gels, monomers and crosslinkers are mixed on the ground before pumping into the lost circulation formation and then performing their plugging role under certain conditions. The polyacrylamide gel HTCMG developed by Jiang et al. can form a gel in the temperature range of 80–150 °C with adjustable gelation time, effectively plugging a 3 mm fracture at 150 °C with a pressure-bearing capacity of 9.8 MPa [33]. Inorganic gel LCMs mainly include mixed slurries (such as cement, gypsum, and lime), with cement being the most commonly used material. They provide high pressure-bearing capacity after plugging the lost circulation layer and have a simple operation process. When combined with a variety of accelerators and retarders, the application scope of inorganic gel LCMs is significantly broadened. Liu et al. suggested that polymers widely used in the construction industry can be applied in the drilling industry, and achieved fast gelation by mixing with non-aqueous drilling fluids (NAFs) [34]. Johnson and Quinn et al. developed a shear-sensitive plugging fluid (SSPF) from the inverse emulsion polymerization of crosslinkers in the oil phase and high-concentration polysaccharide polymers in the aqueous phase [35,36]. This formed a high-strength gel (similar to a plastic solid) from the SSPF that displayed excellent plugging performance.

### 2.3. Research Progress of Gels for Hydraulic Fracturing

Hydraulic fracturing is a common technique for improving recovery in oil fields. Here, the key is to select an appropriate fracturing fluid, and polymer gel fracturing fluids are currently the most prevalent type [37]. The addition of polymer gels increases the viscosity of the fracturing fluid, improves the ability of the fracturing fluid to transport proppant, and reduces filtration losses of the fracturing fluid. Biopolymer and synthetic polymer fracturing fluids are the most common types of polymer fracturing fluids used for hydraulic fracturing. Biopolymers are used as thickeners for acid-fracturing fluids.

Guar gum (GG) is a high-molecular-weight biopolymer consisting of a mannose backbone and a galactose side chain and has been commonly used in oilfield fracturing fluid systems for a long time, as it increases the viscosity and reduces the loss of fracturing fluid. Wang et al. compared the microstructure and viscoelasticity of hydroxypropyl guar gum (HPG) and carboxymethyl guar gum (CMG), revealing that HPG has higher fiber density, better suspension ability, and slower settling velocity compared to CMG [38]. Song et al. researched the rheological properties of GG and prepared a novel fluorinated hydrophobic associating cationic guar gum gel (FCGG) from the reaction of GG and cationic guar gum (CGG) [39]. Compared with conventional fracturing gels, FCGG displayed improved shear and heat resistance (85 °C), rendering it a good prospect for application in oil fields.

In addition to crosslinked gel systems, physical crosslinked gels based on intermolecular non-covalent bonding interactions have been extensively applied in fracturing fluid systems. Based on the formation of hydrogen bonds between the titanium dioxide (TiO_2_) surface and the hydroxyl groups on HPG, Hurnaus et al. successfully increased the viscosity of the HPG gel solution by a factor of 25 [40]. This was achieved via the crosslinking reaction between TiO_2_ nanoparticles (produced by the hydrolysis of Ti complexes) and HPG. Similarly, Jiang et al. employed micelle polymerization to synthesize a supramolecular gel fracturing fluid with a temperature resistance of 160 °C, good shear recovery, and little residue after gel breaking, rendering it a promising candidate for applications in oil fields [41].

Currently, GG fracturing fluid is the most commonly used fracturing fluid, with extensive on-site application effects. However, the formulation is complex and the on-site preparation process is tedious. Moreover, GG fracturing fluid always leaves a high amount of water-insoluble residue upon breaking, failing to fulfill the requirements of ultra-high temperature fracturing. Although synthetic polymer gel fracturing fluids can overcome the challenges of fracturing in ultra-high temperature wells, they suffer from poor shear dilution performance, high frictional resistance, and low conductivity in proppant-filled layers.

## 3. Research Progress on Improving the Temperature Resistance of Gels and Their Applications

With the continuous depletion of medium and shallow oil and gas resources, deep and ultra-deep oil and gas resources are gaining increasing attention. However, during the drilling process in these deep and ultra-deep formations, high temperature is one of the main challenges for the drilling fluid [42]. When used for oil and gas drilling, polymer gel materials are prone to chain breakage and degradation under high-temperature conditions, which results in reduced gel strength and affects the application performance [43]. The main reason for these problems is that dissolved oxygen in the polymer gel (or residual initiator) has a great influence on gel performance under high temperatures. This is due to the presence of the oxygen/residual initiator, where the main chain of the gel is prone to chain oxidation reactions under high temperatures and breaks at the weak bond where peroxide is generated, resulting in gel destabilization [44]. In addition, under high temperatures, the methylene group of the crosslinked polymer gel is subjected to nucleophilic attack by the protonated solvent (water) and breaks to form a hydroxyl group on one end and an amide on the other end. When the protonated solvent attacks the carbonyl carbon of the amide, the amide breaks into primary amine and carboxylic acid, while the nucleophilic attack of the protonated solvent (water) on the carbonyl carbon of the side-chain amide generates carboxylic acid and ammonia [45]. The breakage and hydrolysis of these groups destroy the three-dimensional network structure of the gel. Extensive studies were conducted on improving the high-temperature resistance of polymer gels, with the main strategies including optimizing the macromolecular chain structure of polymers, optimizing the crosslinker types, and introducing high-temperature resistant additives into the gel components.

**Figure 3:**
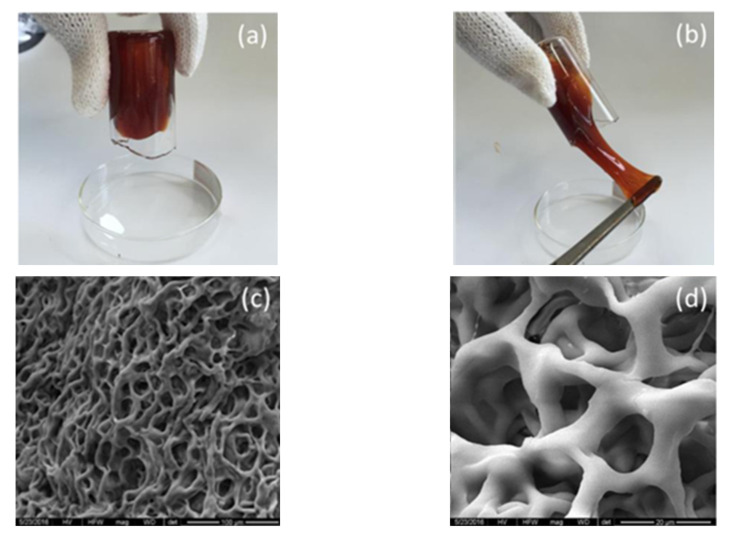
(**a**,**b**) Digital photographs and (**c**,**d**) microstructure images of the gel synthesized from the crosslinking reaction of AM/AA/AMPS and OC-3 [47].

Commonly used polymers in oilfields (such as polyacrylamide, starch, GG, and xanthan gum) are prone to chain breakage and are difficult to degrade at high temperatures. Here, introducing cyclic structures (e.g., a benzene ring) or temperature-resistant groups (e.g., sulfonic acid groups) into the molecular chains of polymers through grafting or copolymerization can significantly improve their temperature resistance, enhancing the high-temperature stability of the gels. Long et al. prepared a high-temperature resistant pre-crosslinked gel system through polymeric crosslinking of acrylamide (AM) with N-vinylpyrrolidone (NVP)- and divinylbenzene (DVB)-containing cyclic structures [46]. The gel strength remained stable after aging at 130 °C for 90 d. Chen et al. prepared an underground crosslinked gel system with good thermal stability and adjustable gel strength by reacting ternary polymer AM/AA/AMPS with OC-3, which is a novel crosslinker containing hydroxyl groups and phenolic rings (Figure 3) [47]. The size of the gel network structure was approximately 10 μm (as determined by microscopy), and the system was free from dehydration after standing for 5 months at 150 °C. Although current research on the optimization of the polymer molecular structure is systematic and in-depth, since polymer gels contain multiple components, research on enhancing the high-temperature stability of gels by simply improving the temperature resistance of polymer molecules has reached a bottleneck.

Crosslinkers are a necessary component of crosslinked polymer gels, and commonly used examples include organic phenolic and metal ion crosslinkers. Chang et al. prepared a polyacrylamide gel by crosslinking resorcinol with formaldehyde [48]. Although the gel exhibited a temperature resistance of up to 120 °C, it was overly sensitive to salinity and acidity. Zhuang et al. performed resorcinol sulfonate methylation, which significantly improved the salt, acid, and alkali resistance of the corresponding gels [49]. When subjected to high temperatures, metal ion crosslinked gels are prone to hydrolysis, resulting in gel dehydration. To solve this problem, Zhang et al. prepared a composite crosslinked polyacrylamide gel system using both Cr^3+^ and phenolic resin as the crosslinkers [50]. The presence of this double-bond network in the system effectively inhibited high-temperature dehydration of the metal crosslinked structure. Accordingly, the developed gel exhibited significantly improved temperature resistance and remained stable, even after aging at 140 °C for 120 d.

Nanoparticles have the advantages of small size, large specific surface area, and high surface activity. Moreover, they can be added to gel formulation as a performance regulator to improve the crosslinking degree between polymer molecules and densify the network structure, optimizing temperature resistance. Almoshin et al. prepared an underground crosslinked gel system by reacting zirconia/graphene nanocomposites with low-molecular-weight polyacrylamide [51]. They demonstrated that the addition of zirconia/graphene resulted in the formation of a smooth surface honeycomb microscopic network structure, allowing the gel to bind internal water molecules at high temperatures. Nano-silica (SiO_2_) is another commonly used solid additive in gels. On the one hand, SiO_2_ fills the voids in the three-dimensional network structure of the gel to enhance the strength of the gel skeleton. On the other hand, SiO_2_ can bond with the hydrophilic groups on the gel skeleton through hydrogen bonding to densify the gel network structure. Jia et al. introduced nano-SiO_2_ into a composite gel lost circulation control agent, and the thermal stability of the gel was enhanced by up to 38% [52]. However, the filling nanomaterials for polymer gels are mainly based on the principle of physical toughening to improve their high-temperature stability, not for essentially solving the high-temperature failure problem of polymer gels.

## 4. Research Progress on High Temperature Resistant Gel Temporary Plugging Materials and Their Applications

Polymer gel temporary plugging materials are mainly used for reservoir protection after completion drilling and completion, fracture steering, profile control, and temporary plugging purposes. Although they have currently achieved a good pressure-bearing temporary plugging effect, they generally have insufficient high-temperature and long-term stability [53]. Table 3 presents a comparison of the different types of high-temperature resistant gel temporary plugging materials. Although these achieve certain application effects, some problems remain. For example, despite their good high-temperature resistance, their stability is weakened after being injected into the formation; hence, they fail to provide long-term effective plugging [54]. High-temperature resistant gel temporary plugging materials can form effective temporary plugging in fractured formations. However, they have poor breaking properties and cannot break in a controlled period, causing certain damage to the reservoir. Moreover, compared with conventional gel materials, high-temperature resistant gel plugging materials are costly and require complex processing. Therefore, there is an urgent need to develop high-temperature resistant gel temporary plugging agents with self-breaking properties and controllable stability [55]. Currently, an effective measure to solve the above problem is to prepare high-temperature resistant functional materials using high-temperature resistant monomers and natural macromolecular functional monomers under the combined action of crosslinkers and initiators and simultaneously prepare self-degradable microcapsule gel breakers with controllable gel breaking time [56]. In use, the microcapsule gel breaker is injected into the formation with the high-temperature resistant gel and starts to release slowly after a period of plugging by the gel. Hence, the gel gradually breaks and achieves temporary plugging of the formation.

Polymer gels have strong temperature and salt resistance, a significant plugging effect, and relatively large resistance and residual resistance coefficients. Moreover, polymer gels perform water shutoff and oil displacement functions simultaneously and are small, allowing them to disperse easily in aqueous solutions to form a suspension. Urdahl et al. developed a polymer gel for temporarily plugging the formation with smooth flowback, which effectively protected the reservoir and created good economic benefits [61]. This gel was successfully applied in the Ekofisk oilfield with simple and convenient on-site formulation and preparation. Cole and Evans developed an environmentally friendly polymer gel temporary plugging agent consisting of modified hydroxyethyl cellulose, a low-toxic metal crosslinker, and a pH regulator [62,63]. This temporary plugging agent exhibited good pumping performance and temperature and salt resistance, fulfilling the requirements of well intervention and completion operations in formations with different pressure coefficients. This temporary plugging agent was successfully applied in 57 wells in the Gulf oilfields and has exhibited good lost circulation prevention and reservoir protection effects.

Chang et al. developed a novel hydroxyethyl cellulose crosslinked system for balancing high-permeability, or high-temperature reservoirs, which mainly consisted of hydroxyethyl cellulose, zirconium salt, and a pH regulator [48]. This system can be formulated with high-concentration saline and can effectively control lost circulation and protect the reservoir in formations with the permeability of 2D and temperatures of 143 °C. This enables a permeability recovery rate of 80–100% and has been successfully applied in the Louisiana, California, and Gulf oil fields. Khater et al. developed a low-damage gel temporary plugging agent for horizontal open hole completion, which is mainly composed of modified hydroxyethyl cellulose and a crosslinker [64]. This temporary plugging agent system is a cost-effective technical method for high water-bearing wells and allows a permeability recovery rate of 99.9% after gel breaking by acid. This system was also applied successfully in oilfields.

Wilsons developed a high-temperature resistant solid-free gel temporary plugging agent that is applicable in operations such as workover, perforation, and completion testing [65]. The temporary plugging agent is mainly composed of a polymer and a metal crosslinker and is prepared with NaBr brine. It is a viscous liquid with good flowability under normal temperatures and pressures, and it crosslinks rapidly into a high-strength temporary plugging gel under high temperatures (176 °C). This temporary plugging agent effectively prevents lost circulation with the low pressure required for plugging removal and allows smooth and natural flowback during operation. Jia et al. proposed a self-matching lost circulation control technology for workover operations and developed a polymer plugging material that breaks automatically at a certain temperature [10]. The characteristics of this system include high-temperature resistance, good viscoelasticity, high deformation degree, high strength, and water-swelling properties. In addition, in combination with other auxiliary temporary plugging materials, a composite plugging system integrating gel, self-degrading material, oil-soluble resin, and microfoam was formulated, which quickly formed a plugging zone in the lost circulation formation and effectively reduced the lost circulation of workover fluid.

For high-temperature and high-salinity reservoirs, polymer gel temporary plugging agents have limitations, such as insufficient strength, uncontrollable temporary plugging time, a complicated recycling process, and a propensity for causing pollution. Therefore, to fulfill the requirements of temporary plugging in high-temperature and high-salinity reservoirs and to enhance the recovery of crude oil, the performance of existing temporary plugging agents must be significantly improved and perfected. Accordingly, the development of novel polymer gel temporary plugging agents that are low-cost, environmentally friendly, long-lasting, and that can be applied to different reservoir conditions is a realistic problem that requires an urgent solution.

## 5. Research Progress on Gel Breaking Methods and Applications

Current gel breaking methods include environmental self-breaking, oxidizing breaking, and acid-etching breaking [66]. However, there are inherent problems, including poor controllability of the self-breaking time. Furthermore, oxidizing/acid-etching breaking requires subsequent auxiliary procedures. The technology of self-breaking initiated by capsules is the key to realizing controllable timing of the downhole breaking of gel temporary plugging materials [8]. Commonly used gel breakers are persulfate-based oxidants, such as ammonium persulfate, sodium persulfate, and potassium persulfate. Capsule gel breakers have delayed release properties and can be adapted for use in medium and deep well environments. There are three main release mechanisms: extrusion crushing release, osmotic release, and chemical dissolution release [67]. The selection of the capsule core and capsule coating is the key to expanding the application scope of capsule gel breakers. Current capsule gel breakers are mainly coated oxidants, which are predominantly used for fracturing fluid breaking and flowback [68]. They have a short application time (120 °C, 5 d) and poor long-term stability under high-temperature conditions. Table 4 summarizes the core materials and technical characteristics of the different types of capsule gel breakers.

### 5.1. Oxidizing Gel Breaking Method

Oxidizing gel breaking mainly breaks the polymer backbone through free radicals to break the polymer fracturing fluid. Under the action of oxidants, the molecular weight of the polymer and the gel viscosity decrease, facilitating the flowback of the fracturing fluid [73]. The commonly used oxidants include peroxides, bromates, chlorites, and persulfates (such as ammonium persulfate, sodium persulfate, and potassium persulfate) which are most commonly used as gel breakers. Although the breaking of GG and polyacrylamide polymer gel can be achieved by oxidants, persulfates are highly active and will cause premature breaking of the polymer at temperatures over 90 °C. Moreover, persulfates hydrolyze in water to generate persulfate anions, causing premature breaking of the polymer gels.

The reactivity of persulfates is closely related to temperature. For example, the activity of ammonium persulfate is low at temperatures below 50 °C, which is not sufficient to produce enough free radicals. Hence, it exhibits a poor gel-breaking effect [74]. At temperatures above 100 °C, ammonium persulfate reacts with the fracturing fluid rapidly, leading to premature breaking of the fracturing fluid and a rapid reduction in viscosity. This affects the fracturing fluid’s performance of carrying proppants and is likely to cause desanding and sand plugging, possibly resulting in failure of the whole fracturing process. In practical applications, the appropriate gel breaker should be selected according to the formation temperature and actual conditions in the oil and gas field [75]. In addition, oxidant gel breakers can react with transport pipes, the formation matrix, and substances such as hydrocarbons, generating substances that are not compatible with the reservoir, causing serious damage to the reservoir. By adding reducing agents, the application temperature of oxidant gel breakers can be reduced to achieve low-temperature gel breaking.

Oxidant gel breakers are very commonly used, and the gel breaking speed is dependent on the decomposition rate of the oxidant. Oxidants decompose relatively slowly in low temperatures and excessively quickly in high temperatures, resulting in an uncontrollable gel breaking rate. Hence, this risks desanding (due to fast gel breaking), meaning they fail to fulfill the requirements of fracturing fluid gel breaking. Here, adding a reducing agent to form an oxidation-reduction system or preparing microcapsules to encapsulate the oxidant can be applied to resolve the problem. Moreover, the gel-breaking speed can be retarded by the slow-release feature of microcapsules.

### 5.2. Enzyme Gel Breaking Method

Enzyme gel breakers are biological materials that are composed of long chains of amino acids bound together by peptide bonds, and their action process is environmentally friendly [76]. Enzymes were first used in fracturing fluids in the 1990s. Theoretically, enzymes have superior performance compared to oxidant gel breakers due to their inherent specificity and “unlimited” polymer degradation activity. Moreover, enzymes have exhibited excellent properties when acting as catalysts to accelerate chemical reactions [77]. The corresponding gel-breaking speed mainly depends on enzyme activity, and enzyme gel breakers require the pH value to be maintained at a low level. Although most enzymes need to be modified for gel-breaking purposes, their structures remain unchanged during the initiation of the reaction.

Enzymes applied in the fracturing process currently include cellulases, hemicellulases, pectinases, and amylases [78]. Unlike oxidants that degrade polymer chains randomly, each type of enzyme degrades specific bonds in the polymer backbone. Moreover, starch molecules can be grafted with ethylene-based monomers through polymerization, forming polymers with a large number of functional groups (such as hydrophilic carboxyl and amino groups) in the side chains. Starch-graft polymers with starch as the main chain and ethylene-based polymers containing a large number of hydrophilic groups as side chains can be used as highly absorbent resins with unique water-swelling and biodegradation properties. In addition, the resin strength can be regulated by adjusting the degree of crosslinking, making them a potential temporary plugging material for drilling fluids.

Employing the enzymatic decomposition method, Aggarwal et al. investigated the biodegradation properties of a polyacrylonitrile polymer containing starch components [79]. The results indicated that the starch component contained in the polymer could be broken down rapidly by amylase and glucoamylase. Brannon et al. proposed that if sulfate could attack the β-1,4 glycosidic bond in the mannose backbone or the β-1,6 glycosidic bond in the galactose side substituent, the molecular weight of guanidine gum polymers would be rapidly reduced, achieving rapid viscosity reduction [80]. Caulfield et al. revealed that the degradation was more pronounced in the presence of potassium persulfate than in the presence of hydrogen peroxide [81].

Using enzymes as gel breakers has many advantages. First, enzymes act as catalysts in the system and are not consumed in the breaking process. Therefore, the degradation of polymers can be maintained at a high level. Second, there are no side reactions and there is less damage to the well. However, under high-temperature conditions, enzymes lose their activity. Therefore, they can only be applied to systems with a temperature below 60 °C and a pH in the range of 3–8. Accordingly, for most temporary plugging agents, enzyme gel breakers do not meet the requirements.

### 5.3. Acid Gel Breaking Method

Acids have been used to degrade drilling fluid gels for an extensive period. They dissolve acid-soluble matter in the drilling fluid filter cake, causing degradation and gel breaking. Acids are also very reactive, reacting rapidly with the acid-soluble bridging particles (CaCO_3_) in the filter cake when injected into the reservoir. The rate of acid dissolution is 10 to 20 times faster than the acid-catalyzed hydrolysis of polymers in the filter cake. In addition, acids have low reaction specificity [82]. For example, they can react with production casings and be consumed, causing corrosion to the equipment. Potential acids, such as organic esters (including methyl formate, ethyl acetate, and triethyl phosphate) and compounds (such as trichlorotoluene, dichlorotoluene, and chlorobenzene) can release acids under high-temperature conditions. This causes acid-catalyzed hydrolytic breaking of acetal bonds in plant gums and their derivatives, as well as cellulose and its derivatives. They are applicable to oil and gas formations of 93 °C. Weak acids are generally used in borate-crosslinked gels. Borate-crosslinked gels are formed under alkaline pH conditions [19]. Acids release H+ through hydrolysis reactions, lowering the pH of the fracturing fluid and enabling the decrosslinking of crosslinked polymer chains, resulting in a decrease in viscosity. However, the use of acids does not degrade the polymer backbone.

The role of acid gel breakers is to gradually change the pH of the fracturing fluid to within a certain range, where it becomes unstable and hydrolysis or chemical decomposition of polymers occurs. Most acids used as gel breakers are slowly dissolving organic acids that affect the solution pH when dissolving. The rate of this pH change is determined by the initial buffer concentration, reservoir temperature, and acid concentration. Due to their property change (e.g., consumed by acid-soluble minerals in reservoir rocks), acids are not commonly used as gel breakers. In the actual breaking process of water-based fracturing fluids, the application of acids is limited by their high cost, control difficulties, and poor compatibility.

## 6. Summary

The application performance of existing gel water shutoff and profile control systems is poor in high-temperature and high-salinity oil fields. To improve the high-temperature stability of polymer gel materials, research should focus on the preparation of high-temperature resistant polymers and crosslinkers, as well as filling the gel structure with high-temperature resistant solid materials. For the former, in-depth research and development of novel high-temperature resistant polymers and crosslinkers from the perspective of molecular design are required. For the latter, studies on the action mechanism of solid particles need to be strengthened to provide a reference for further preferential selection of solid filling materials with excellent performance. To meet the requirements of temporary plugging in high-temperature and high-salinity reservoirs and to enhance the recovery of crude oil, the performance of existing temporary plugging agents must be improved. In addition, research into novel polymer gel temporary plugging agents with low cost, environmental friendliness, and long-lasting adaptability to different reservoir conditions is urgently needed.

The most commonly used methods for gel breaking are biological and chemical. Enzymes and oxidants are the most frequently used, followed by acids. Oxidants break the polymer backbone rapidly and effectively to achieve gel breaking. The higher the temperature, the more reactive the oxidants are, and the gel breaking is quicker and more complete. However, gel breaking by oxidants is difficult in the fracturing process in low-temperature oil and gas formations. At low temperatures, enzyme gel breakers are more effective. Moreover, with the continuous progress of science and technology, an increasing number of enzymes are exceeding traditional pH and temperature limits and have started playing an important role in gel breaking. In comparison, acids are not commonly used as gel breakers. The selection of gel breakers determines the flowback effect of the broken fluid and plays a decisive role in the operation effect of oilfields.

## Figures and Tables

**Table 1:** Research progress and application status of gel technologies developed by oil service companies and institutes in different countries.

Company/Institute	Technology	Core Materials	Technology Characteristics
Schlumberger	CACP chemical crosslinking lost circulation control technology [2]	Crosslinked polymer, biomacromolecule, crosslinker, retarder, fiber	Suitable for water-based and oil-based drilling fluids, with rapid downhole gelation, strong gel structure, good water-plugging effect, and resistance to thermal degradation
Baker Hughes	Crosslinking lost circulation control technology [13]	Crosslinked polymer, acid-soluble bridging particle, organic crosslinker, retarder	Crosslinking time can be tuned by adjusting the crosslinker, retarder, and downhole temperature; self-breaking occurs in long-term high-temperature environments
Halliburton	Chemical plugging technology CS-LCM [14]	Tackifier, clay, reactant, activator	Direct gelation feature, ability to plug 31 mm circular hole, pressure bearing capacity of 6.8 MPa, good pumping performance, 100% acid soluble
Sinopec Research Institute	Downhole crosslinked gel SF-1 [15]	Chitosan, cationic polyacrylamide, HDL curing material	Temperature resistance of 120 °C, curing upon contact with chemical consolidation plugging slurry, compressive strength of up to 15 MPa
Sinopec Research Institute	Controllable smart crosslinked gel [16]	Polyacrylamide, inorganic Cr^3+^ crosslinker	Delayed crosslinking achieved by coating crosslinker with polyvinyl alcohol, crosslinking time of 9–12 h, temperature resistance of 60–90 °C
Northwest Oil Field Company	Downhole crosslinked gel [17]	Thiourea, temperature resistant polymer, hexamethylenetetramine, methylparaben, oxalic acid, fiber, baryte	Temperature resistance of 120 °C, pressure-bearing capacity of 2.5–11 MPa for 2–5 mm fractures, stable for 10 d under high temperature, automatic flowback after 15 d
Southwest Petroleum University	Special Gel ZND [18]	Polymer formed by hydrophobic association of macromolecules	Excellent shear dilution performance and anti-dilution properties, not suitable for high-temperature high-salinity environments

**Table 2:** Research progress of gels for profile control and water shutoff.

Gel type	Typical Products	Gelation Mechanism	Advantages	Disadvantages
In situ crosslinked gel [19]	monomer crosslinked gels, organic crosslinked gels, and metal crosslinked gels	Free radical-initiated polymerization, crosslinker interactions to form covalent and ionic bonds	Low initial viscosity, high mineralization resistance, good plugging effect in high permeability channels, high gel strength	Prone to shear influence, significant negative impact on low permeability layers, sensitive to oilfield environments
Ground pre-crosslinked gel [20]	Pre-crosslinked gel particles, polymer gel microspheres	Gel synthesized from monomers, dried and ground into particles; resulting particles are nanosized	Good thermal stability and low sensitivity to mineralization; improved viscoelasticity of polymer gel microspheres	Microsphere particles are small and not suitable for plugging in high permeability and fractured formations

**Table 3:** Comparison of different types of high-temperature resistant gel temporary plugging materials.

Material System	Core Materials	Action Mechanism	Characteristics
High-temperature thickening crosslinked temporary plugging agent system [53]	Filler SDC-A, crosslinker SDC-B, ammonium persulfate, and gel CY-A27	Ammonium persulfate is the gel breaker, forming an emulsion in the early stage after gel breaking	High-temperature resistance, high degree of gel breaking, strong pressure-bearing capacity of the temporary plugging layer
HPAM/phenolic/urea-formaldehyde crosslinked polymer gel temporary plugging agent [13]	HPAM, phenolic, and urea-formaldehyde crosslinked polymer	HPAM/phenolic/urea-formaldehyde crosslinked polymer compounding to form a temporary gel-plugging agent system	Gel temporary plugging agent is prepared at 75 °C for temporary plugging purposes in high-temperature reservoirs below 120 °C.
High-temperature resistant curable elastic blend gel temporary plugging agent system [57]	Acrylamide copolymer, polyethyleneimine, toughening material sodium-based montmorillonite, and thiourea	The gel is produced from the reaction of acrylamide copolymer, crosslinker polyethyleneimine, toughening material sodium-based montmorillonite, and thiourea.	The system is stable without dehydration for more than 10 d at 130–160 °C.
Degradable preformed particle gel (DPPG) temporary plugging agent system [58]	Acrylamide, 2-acrylamido-2-methylpropanesulfonic acid, PEGDA-250, and KPS	Introduction of acid-resistant functional groups and self-degradable crosslinked structures into the composition of conventional PPG	Good water-swelling property, self-degradation to fluid in acidic solutions, little damage to the reservoir.
Self-breaking gel temporary plugging system [59]	Acrylamide, monomer GXDF, crosslinker JF, and initiator YF	Formed from reactions between self-hydration degradable polymers and other auxiliary temporary plugging materials	Pressure-bearing capacity of up to 15 MPa, plugging efficiency of >90%, and permeability recovery rate of >90%.
High-temperature resistant copolymer gel temporary plugging system [60]	Acrylamide, 2-acrylamido-2-methylpropanesulfonic acid, terpolymer, crosslinker, and initiator	Achieving plugging of different lost circulation formations via tuning concentrations of terpolymer and crosslinker	The system forms a stable and continuous three-dimensional network structure at high temperatures (120–200 °C) with excellent long-term thermal stability.

**Table 4:** Research progress on capsule gel breakers.

Company/Institute	Technology	Core Materials	Technical Characteristics
Beijing Chemrein Energy Science Co., Ltd.	Controllable capsule gel breaker and its preparation method [69]	Temperature-resistant micro-permeable capsule core is a curing agent and epoxy resin; temperature-resistant waterproof polymer capsule coating is polyether, polyolefin copolymer, and polyester acid copolymer	Suitable for a variety of gel breaking objects, strong gel breaking ability; applicable to gel-breaking temperatures of 0–120 °C with good gel-breaking ability.
Yangzhou University	Preparation and performance study of microcapsules with controllable release of ammonium persulfate [70]	Preparation of slow-release microcapsules by in situ polymerization with solid ammonium persulfate particles as the core material and polypyrrole as the coating material	The release time can be regulated by adjusting either the thickness of the coating, the concentration of glycerol monomer in the coating, or the temperature.
Jidong Oilfield Company	Preparation of core-shell delayed gel breaker by sol-gel method [71]	With tetraethoxysilane (TEOS) as the precursor, core-shell delayed gel breaker with silica as the coating and ammonium persulfate as the core is prepared by sol-gel method	Delayed gel breaker fulfills the requirements of underbalanced completion operations at 120 °C and delayed gel breaking for 5 d.
Southwest Petroleum University	Preparation method of potassium persulfate microcapsule gel breaker [14]	Oxidant potassium persulfate as the core, gelatin as capsule coating solution, and sorbitan monooleate as the stabilizer	microcapsule gel breaker has advantages of good sphericity, uniform and controllable particle size distribution, high effective content, and slow release. Can be used for gel breaking of water-based fracturing fluids in oilfields.
China National Petroleum Corporation	Capsule gel breaker and its preparation method [72]	Encapsulation of solid gel breakers with amphiphilic modified starch or amphiphilic modified cellulose derivative grafted acrylic resin-ethylene copolymer as capsule coating	Prolonged release time and controllable slow release time of capsule gel breakers, achieving self-breaking of gels at the target time to complete gel flowback

## Data Availability

Not applicable.
